# Problems in care and avoidability of death after discharge from intensive care: a multi-centre retrospective case record review study

**DOI:** 10.1186/s13054-020-03420-5

**Published:** 2021-01-06

**Authors:** Sarah Vollam, Owen Gustafson, J. Duncan Young, Benjamin Attwood, Liza Keating, Peter Watkinson

**Affiliations:** 1 Nuffield Department of Clinical Neurosciences, University of Oxford, Level 6, West Wing, John Radcliffe Hospital, Oxford, OX3 9DU UK; 2 National Institute for Health Research Biomedical Research Centre, Oxford, UK; 3 Therapies Clinical Service Unit, Oxford University Hospitals NHS Foundation Trust, Oxford, UK; 4 Adult Intensive Care Unit, South Warwickshire NHS Foundation Trust, Warwick, UK; 5 Adult Intensive Care Unit, Royal Berkshire NHS Foundation Trust, Reading, UK

**Keywords:** In-hospital death, Avoidable harm, Critically ill

## Abstract

**Background:**

Over 138,000 patients are discharged to hospital wards from intensive care units (ICUs) in England, Wales and Northern Ireland annually. More than 8000 die before leaving hospital. In hospital-wide populations, 6.7–18% of deaths have some degree of avoidability. For patients discharged from ICU, neither the proportion of avoidable deaths nor the reasons underlying avoidability have been determined. We undertook a retrospective case record review within the REFLECT study, examining how post-ICU ward care might be improved.

**Methods:**

A multi-centre retrospective case record review of 300 consecutive post-ICU in-hospital deaths, between January 2015 and March 2018, in 3 English hospitals. Trained multi-professional researchers assessed the degree to which each death was avoidable and determined care problems using the established Structured Judgement Review method.

**Results:**

Agreement between reviewers was good (weighted Kappa 0.77, 95% CI 0.64–0.88). Discharge from an ICU for end-of-life care occurred in 50/300 patients. Of the remaining 250 patients, death was probably avoidable in 20 (8%, 95% CI 5.0–12.1) and had some degree of avoidability in 65 (26%, 95% CI 20.7–31.9). Common problems included out-of-hours discharge from ICU (168/250, 67.2%), suboptimal rehabilitation (167/241, 69.3%), absent nutritional planning (76/185, 41.1%) and incomplete sepsis management (50/150, 33.3%).

**Conclusions:**

The proportion of deaths in hospital with some degree of avoidability is higher in patients discharged from an ICU than reported in hospital-wide populations. Extrapolating our findings suggests around 550 probably avoidable deaths occur annually in hospital following ICU discharge in England, Wales and Northern Ireland. This avoidability occurs in an elderly frail population with complex needs that current strategies struggle to meet. Problems in post-ICU care are rectifiable but multi-disciplinary.

*Trial Registration*: ISRCTN14658054.

## Background

For patients discharged alive from an intensive care unit (ICU), the subsequent in-hospital days are high risk. Post-ICU in-hospital mortality rates are 4–13% worldwide [1–3] and 6.6% in England and Wales [4]. Around a third of in-hospital mortality in those treated on an ICU occurs between ICU and hospital discharge.

Risk factors for post-ICU in-hospital mortality identifiable whilst in ICU have been investigated using ICU databases [1–3, 5, 6]. These studies show older, sicker patients are more at risk. However, there is little work identifying either what proportion of these deaths are avoidable or how post-ICU care could be changed to decrease mortality. Recent studies attempting to improve mobility or nutrition post-ICU have not changed outcomes [7, 8] emphasising the need to better understand where successful interventions could be directed.

Hogan et al. used retrospective case record review (RCRR) to investigate preventability of deaths in English hospitals [9]. This work established the RCRR methodology for mortality review, since refined into the Structured Judgement Review method (SJR) [10]. The SJR approach standardises critical assessment of care delivery, splitting a hospital stay into defined care periods. The SJR has been used internationally [11] and implemented throughout NHS hospitals in England [12]. It is advocated as the mortality review method for all UK NHS ICUs [13]. However, to the best of our knowledge this approach has not previously been used to examine deaths following ICU discharge.

This work is part of the REFLECT project, with the overall aim of developing a multi-component intervention to reduce post-ICU in-hospital mortality [14]. The primary aim of this study was to quantify the avoidability of deaths in patients on hospital wards following an ICU admission, using RCRR methodology. We also report care areas that could be changed to improve outcomes in this vulnerable patient group.

## Methods

We report our study according to the STROBE statement [15]. We obtained ethical approval (Wales REC 4 reference: 17/WA/0139) and Confidentiality Advisory Group support (reference: 17/CAG/0063). We published the protocol [14] and registered the study (ISRCTN14658054).

### Setting

We conducted SJRs in three UK hospitals in separate NHS trusts, in adjoining regions: a large tertiary referral centre, a large university-affiliated district general hospital and a small district general hospital. We selected sites representing different clinical settings both within and outside the ICUs (Additional file 1: Table S1). All sites had nurse-led ICU outreach/follow-up services who visit patients discharged from ICU to the ward.

### Sampling strategy

#### Non-survivors

We pre-specified a sample size of 300 consecutive patients who had died post-ICU in hospital prior to April 2018 in our published protocol [14] based on a previous audit. From this we anticipated around 10% of deaths reviewed would be avoidable and that 300 patients would be available from participating hospitals within approximately three preceding years, allowing us to represent recent practice. We excluded patients with missing medical notes or who were transferred away from study hospitals.

We selected cases using electronic hospital records in reverse chronological order from March 2018 until 300 eligible cases were identified across the three sites. We excluded cases where medical notes were unavailable (e.g. missing) or the record was incomplete (e.g. key documents such as observations charts or sections of medical documentation were missing from the record of care). We reviewed ward care following the first discharge from an ICU.

#### Survivors

A convenience sample of cases was selected from participants interviewed as part of the REFLECT study. Details of the approach are included in the published protocol [14]. We reviewed an equal number of survivor cases as ‘probably avoidable’ deaths to offer a comparison of care.

### Variables

We collected summary data on all patients discharged from each ICU during the study period including age, sex and Clinical Frailty Scale (CFS) prior to hospital admission [16], type of admission (surgical/medical and elective/emergency), Acute Physiology and Chronic Health Evaluation (APACHE II) score on ICU admission [17], ICU length of stay (LOS) and post-ICU ward.

Detailed data on deaths following discharge from ICU were collected using the SJR methodology [18]. The SJR form [12] categorises care into distinct periods: initial management; ongoing management; care during a procedure; end-of-life care; and overall care. For each period, a short narrative account of care is written, including ‘judgement statements’. A quality of care score is assigned to each period on a scale from 1 (very poor care) to 5 (excellent care). Scores for each care period contribute to a final overall score of quality of care, derived from and supported by the judgement statements. Following review of care, an ‘avoidability of death’ judgement score is assigned, considering any problems in care identified as contributing to the outcome. This is a 6-point scale from 6: definitely not avoidable to 1: definitely avoidable.

We piloted the published SJR Case Report Form [12] resulting in minor adjustments to fit the post-ICU cohort. We changed the focus of the ‘initial management’ section from ‘first 24 h in hospital’ to ‘first 24 h following ICU discharge’; added more detailed demographic data; and included focused data capture of pre-identified care issue areas obtained from literature review [3, 19, 20] and a previous local audit. These included discharge from an ICU out-of-hours; mobility and rehabilitation; nutrition; and management of atrial fibrillation (AF) or sepsis. We also collected information on the provision of ICU outreach/follow-up care for each patient.

We recorded when patients were discharged for end-of-life care (EOLC) following ICU discharge and whether death occurred from progression of a chronic disease. We collected additional data on the pre-identified care issues. We recorded problems in care where they were judged to result in harm to a patient. The pre-identified care issues were not defined as a ‘problem in care’ unless they were judged to have resulted in harm to the patient. Data sources and rules (where interpretation was required) were defined for each variable (Additional file 1: Tables S2 and S3).

In line with previous work we defined ‘avoidable deaths’ as those classified ‘probably avoidable’ or greater levels of avoidability (score 1–3). We also calculated a wider ‘relaxed’ definition of preventable deaths (including ‘possibly preventable but not very likely’, score 1–4) [9, 11]. We classified all deaths other than those that were ‘definitely not avoidable’ (score 6) as having some degree of avoidability.

### Data extraction

We extracted summary data for all discharged patients from electronic records. Sources of data included nursing and medical notes, laboratory results, vital signs documentation, therapy documentation, and drug, food and fluid charts. We defined data sources and rules (where interpretation was required) for each variable collected (Additional file 1: Table S3). We extracted data onto paper case report forms. These were then transcribed into a pre-piloted spreadsheet (Microsoft Excel version 16, Microsoft Corporation, Redmond, USA).

We accessed anonymised population descriptive data for patients discharged from an ICU in England, Wales and Northern Ireland over the study period (excluding the study sites) from the Intensive Care National Audit and Research Centre (ICNARC—the national audit covering all general ICUs) case mix programme, to assess the comparability of our sample.

### Bias

Three reviewers (two nurses and a physiotherapist) with clinical experience of both ICU and general wards completed the reviews. All three reviewers attended SJR training, run by the Clinical Governance team in the lead hospital. Reviewers also studied published SJR guidance [21]. To improve agreement, 10 initial cases were dual reviewed and discussed by two reviewers (SV and OG) to develop extraction approaches. Uncertainties and complex cases were discussed and scores agreed. Where uncertainty remained, cases were discussed with an ICU consultant (PW). To assess inter-rator reliability, 15 undiscussed cases were dual reviewed and scores for each care period, overall quality of care and avoidability judgments compared.

### Statistical analyses

The primary outcome measure was the proportion of in-hospital deaths following ICU discharge that were probably avoidable. Secondary outcomes included the proportion of in-hospital deaths following ICU discharge with lesser degrees of avoidability, characteristics of post-ICU non-survivors and survivors, quality of care scores for deaths by avoidability and data on pre-identified care issues and delivery of outreach/follow-up services. Data are presented as mean (95% CIs), median (inter-quartile range) or proportion (%, 95% CI), as appropriate. Confidence intervals of proportions were calculated using the Clopper–Pearson method. Agreement between reviewers was assessed using linear-weighted Cohen’s Kappa with confidence intervals calculated using bootstrapping (10,000 samples). Comparisons of proportion were undertaken using Fisher’s exact test. Analyses were undertaken in R [22].

## Results

### Participants

Between January 2015 and March 2018, 352 of 7434 (4.7%) patients consecutively discharged from the study ICUs died during the same hospital admission. We excluded 52 incomplete (16 records) or unavailable records (36 records). Of the 300 eligible cases, 50 patients were discharged for end-of-life care (Additional file 1: Fig. S1). We reviewed the care of 20 patients who survived to hospital discharge, matching the number of avoidable deaths.

### Descriptive data

Baseline characteristics for study patients were similar to national findings (Table 1). However, the APACHE II scores appeared higher in the study population, suggesting the overall severity of illness and probability of in-hospital death were greater in the study hospitals.

**Table 1:** Patient characteristics

Characteristics	Study data Non-survivors: EOLC *n* = 50	Study data Non-survivors: excluding EOLC *n* = 250	Study data Analyzed survivors *n* = 20	Study data All survivors *n* = 7082	Study data ICU discharges excluding EOLC† *n* = 7332	National CMP†† data ICU discharges excluding EOLC *n* = 437,586
Age median (IQR)	73 (65–82.75)	74 (63.25–80)	66 (55–69.5)	62 (46–73)	62 (47–73)	63 (48–74)
Female n (%)	18 (34.6)	90 (36)	7 (35)	3075 (43.4)	3165 (43.2)	198,319 (45.3)
APACHE II median (IQR)	21 (18–24)	21 (17–26)	16 (14–22)	15 (12–20)	16 (12–20)	13 (10–18)
Admission diagnosis n (%)						
Surgical	14 (28)	112 (44.8)	13 (65)	3529 (49.8)^a^	3641 (49.7)^a^	209,098 (47.8)^d^
Medical	36 (72)	138 (55.2)	7 (35)	3194 (45.1)	3332 (45.4)	228,439 (52.2)
Type of admission n (%)						
Emergency	50 (100)	233 (93.2)	17 (85)	4352 (61.5)^b^	4585 (62.5) ^b^	313,790 (71.7)^d^
Elective	0 (0)	17 (6.8)	3 (15)	1702 (24.0)	1719 (23.4)	123,747 (28.3)
Clinical frailty scale n (%)						
1–4	23 (46)	128 (51.2)	15 (75)	5471 (77.3)^c^	5599 (76.4)^c^	339,919 (77.7)^e^
5	12 (24)	57 (22.8)	3 (15)	1218 (17.2)	1275 (17.4)	73,822 (16.9)
6	5 (10)	44 (17.6)	2 (10)	185 (2.6)	229 (3.1)	17,631 (4.0)
7–9	10 (20)	21 (8.4)	0	105 (1.5)	126 (1.7)	4459 (1.0)
ICU LOS (hours) median (IQR)	72 (48–144)	96 (48–168)	312 (138–534)	72 (48–120)	72 (48–120)	57 (26–120)
Post-ICU LOS (days) median (IQR)	1.5 (0–4)	9 (5–21)	15.5 (6.5–24.5)	8 (4–17)	8 (4–17)	8 (4–16)

*EOLC* end-of-life care, *LOS *length of stay

^†^Excluding 52 ward deaths not included in the review

^††^Case Mix Programme

^a^359 (5%) missing

^b^1028 (14.5%) missing

^c^103 (1.5%) missing

^d^49 (0.01%) missing

^e^1755 (0.4%) missing

Patients discharged from ICU for end-of-life care who died before leaving hospital were numerically more likely to be male medical patients and tended to be older, frailer and with higher APACHE II scores than survivors (Table 1). By the 9th day following discharge 50% of deaths occurred (Additional file 1: Fig. S2).

### Avoidability of death and quality of care

Overall agreement between reviewers was good (weighted Kappa 0.77, 95% CI 0.64–0.88 for all scores combined) (Additional file 1: Table S4). During review of the 300 cases, only two were discussed with a third party (PW), where uncertainties could not be resolved between two reviewers. Death had some degree of avoidability (scoring one to five) in 65/250 (26%, 95% CI 20.7–31.9) of cases (Table 2). For 20 patients (8%, 95% CI 5.0–12.1) death was probably avoidable (more than a 50:50 chance of avoidability). For the more relaxed definition of avoidability, 44 (17.6%, 95% CI 13.1–22.9) patients qualified. Two case vignettes are presented below, illustrating examples of deaths judged to be probably avoidable and possibly avoidable.

**Table 2:** Overall avoidability of death scores

Scale used to judge avoidability of death *n* (%)	Discharges from ICU, excluding EOLC *n* = 250 (% [95% CI])
1. Definitely avoidable	0 (0 [0–1.5])
2. Strong evidence of avoidability	0 (0 [0–1.5])
3. Probably avoidable (more than 50:50)	20 (8) [5.0–12.1])
4. Possibly avoidable but not very likely (less than 50:50)	24 (9.6 [6.2–14.0])
5. Slight evidence of avoidability	21 (8.4 [5.3–12.6])
6. Definitely not avoidable	185 (74 [68.1–79.3])

*EOLC* end-of-life care

**Table:** Vignette 1. Probably avoidable (more than 50:50) and poor care.

An elderly patient was discharged on a weekend evening with a high early warning score after a 1-day elective ICU admission following major intra-abdominal surgery. Their surgery was deemed high risk because of a past history of significant chronic obstructive pulmonary disease (COPD). As the patient was at risk of developing a hospital-acquired respiratory infection, a plan for immediate treatment with antibiotics in the event of respiratory deterioration was decided on by the ICU team. However, this plan was not included in the patient’s ICU discharge document. The patient’s low oxygen saturations worsened from the first post-discharge day but were attributed to fluid overload. Physical examination of the chest was not documented, and mobilisation did not occur. A chest X-ray was taken on the evening of the second day following ICU discharge but was not reported until a specialist respiratory team reviewed the patient on the afternoon of the third post-discharge day. The respiratory team diagnosed hospital-acquired pneumonia, commenced appropriate antibiotics and arranged chest physiotherapy (which had not occurred following ICU discharge). The patient deteriorated further. A decision was made not to escalate treatment, and the patient died from hospital-acquired pneumonia. *Overall judgement *Discharge from an ICU late in the day with continuing physiological abnormalities leading to a high early warning score with inadequate information exchange at ICU discharge contributed to missed subsequent opportunities to prevent or manage hospital acquired pneumonia in a patient at risk for this complication.	

**Table:** Vignette 2. Possibly avoidable but not likely with poor care.

An elderly frail patient with cardiac and other co-morbidities was discharged from ICU during the day, following emergency abdominal surgery. A clear management plan was in place at the time of ICU discharge. They received no medical team review, physiotherapy assessment or critical care follow-up on the first post-ICU ward day. Hypotension was first recognised the next day, leading to an increased early warning score. The hypotension was not addressed in a consultant review, and they were discharged from the critical care outreach service. Worsening hypotension subsequently led to suspicion of abdominal sepsis and the Sepsis Six care bundle complied with, including administration of antibiotics. On the same day they were treated for a suspected myocardial infarction and subsequently developed atrial fibrillation. They continued to deteriorate until instigation of end-of-life care several days later. *Overall judgement* Delay in the initial recognition and management of sepsis and myocardial infarction may have contributed to the outcome; however, the presence of co-morbidities and frailty suggests their death was unlikely to have been preventable.	

We judged 185 deaths to have no avoidability: for 51/185 (27.6%) death was caused by progression of a chronic disease (such as liver failure, chronic respiratory disease or cancer); 14/185 (7.6%) were transitioned to end-of-life care within 24 h of ICU discharge and 5/185 (2.7%) died suddenly within 48 h of ICU discharge without ward-based problems in care. Of the remaining 115 patients: in 64/185 (34.6%) death was considered unavoidable despite having problems in care and 51/185 (27.6%) had no problems in care delivery (Additional file 1: Table S5). A case vignette is presented below, illustrating an example of a death judged to be probably unavoidable.

**Table:** Vignette 3: Slight evidence of avoidability and poor care.

An elderly, very frail patient was discharged from ICU during the day, following a short ICU stay after elective abdominal surgery. There was a clear written handover from ICU including a management plan and ICU follow-up occurred. Over the next 3 days they deteriorated with increasing tachycardia, reducing haemoglobin concentration and abdominal distension. Sepsis was suspected and the Sepsis Six care bundle complied with. Although the symptoms of deterioration were treated, there was no investigation of the underlying cause of this deterioration or sepsis source until the fourth day after ICU discharge when a small bowel perforation was diagnosed with a CT scan. The patient returned to ICU but did not recover. *Overall judgement* There was a significant delay in investigating the underlying cause of deterioration. Despite this, the high level of frailty meant the patient was unlikely to have survived.	

Patients received poor or very poor overall care in 46/65 (70.8%, 95% CI 58.2–81.4) cases where death had some degree of avoidability, in comparison with 16/185 (8.65%, 95% CI 5.02–13.7) cases for patients with no problems in care contributing to death (*p* < 0.001). All cases judged to be probably avoidable were judged to have received poor or very poor care. Care was judged poor or very poor overall in 8/20 (40%, 95% CI 19.1–63.9) cases for survivors (Table 3).

**Table 3:** Overall quality of care scores

Score *n* (%)	Deceased patients with some degree of avoidability *n* = 65	Deceased patients without avoidability *n* = 185	Discharges from ICU, excluding EOLC *n* = 250	Survivors *n* = 20
1. Very poor care	8 (12.3)	3 (1.6)	11 (4.4)	0
2. Poor care	38 (58.5)	13 (7)	51 (20.4)	8 (40)
3. Adequate care	15 (23.1)	55 (29.7)	70 (28)	5 (25)
4. Good care	4 (6.1)	109 (58.9)	113 (45.2)	7 (35)
5. Excellent care	0 (0)	5 (2.7)	5 (2)	0

*EOLC* end-of-life care

### Problems in care

The occurrence of problems in care by care period is shown (Table 4). The frequency of problems in a 24-h period was greatest during the first 24-h following discharge, although problems in care occurred most frequently during the ‘ongoing care’ period, as this period was often long (median between 8 and 14.5 days across the groups).

**Table 4:** Number of problems in care by period following discharge from an ICU

Phase of care *n* (%)	Deceased patients with some degree of avoidability Total problems in care = 189	Deceased patients without avoidability Total problems in care = 90	All discharges from ICU, excluding EOLC Total problems in care = 279	Survivors *n* = 20
First 24 h	43 (22.8)	39 (43.3)	82 (29.4)	13 (33.3)
Procedure	1 (0.5)	2 (2.2)	3 (0.43)	0
Ongoing^a^	132 (69.8)	38 (42.2)	170 (60.9)	26 (66.7)
Perioperative	0	0	0	0
End of life	13 (6.9)	11 (12.2)	24 (8.6)	0

*EOLC* end-of-life care

^a^Median length of stay following the first 24 h after discharge was 9 (IQR 4–18) days for deceased patients with problems in care, 8 (IQR 4–22) days for deceased patients with no problems and 14.5 (IQR 5.5–23.5) days for survivors

Pre-identified care issues were common in post-ICU non-survivors (excluding those discharged for end-of-life care) (Table 5). ICU discharge occurred out-of-hours (after 4 p.m.) for 168/250 (67.2%) patients. In 155/250 (62%) cases, patients were unable to stand and step from bed to chair. Of 241 discharges where bed to chair mobilisation was appropriate, mobilisation did not occur in 167/241 (69.3%) on every day this was deemed possible. A new episode of confirmed or suspected sepsis was documented in 150/250 (60%), of whom 50/150 (33.3%) received the full ‘Sepsis 6′ care bundle [23]. A nutrition plan was not documented in 76/185 (41.1%) patients requiring nutritional support. Pre-identified care issues also occurred frequently in survivors after ICU discharge. Follow-up practitioners reviewed 207/250 (82.8%) patients, 66/207 (31.9%) of whom were discharged from the follow-up service on the first post-ICU day.

**Table 5:** Pre-identified care issues

Problems in care	Deceased patients with some avoidability *n* = 65	Deceased patients without avoidability *n* = 185	All discharges from ICU, excluding EOLC *n* = 250	Survivors *n* = 20
Discharged *n* (%)				
16:00–08:59	50 (76.9)	118 (63.8)	168 (67.2)	14 (70)
18:00–08:59	36 (55.4)	76 (41.1)	112 (44.8)	8 (40)
22:00–08:59	8 (12.3)	38 (20.5)	46 (18.4)	2 (10)
Mobility				
Unable to stand and step from bed to chair on ICU discharge *n* (%)	39 (60.0)	116 (62.7)	155 (62.0)	6 (30)
Not mobilised to a chair *n* (%)	46 (73.0) (*n* = 63^b^)	121 (68.0) (*n* = 178^b^)	167 (69.3) (*n* = 241)	7 (35) (*n = *20)
Not mobilised away from bed *n* (%)	42 (84) (*n* = 50^b^)	106 (73.6) (*n* = 144^b^)	148 (76.3) (*n* = 194^b^)	11 (61) (*n *= 18^b^)
Atrial fibrillation				
New diagnosis *n* (%)	9 (13.8)	31 (16.8)	40 (16.0)	1 (5)
Initial management assessed as not appropriate *n* (%)	5 (55.6)	7 (22.5)	12 (30)	0 (0)
No investigation of underlying cause *n* (%)	6 (66.6)	11 (35.5)	17 (42.5)	1 (100)
Sepsis				
Diagnosis/suspicion *n* (%)	43 (66.2)	107 (57.8)	150 (60)	4 (20)
Sepsis 6 not completed *n* (%)	19 (44.2)	31 (29)	50 (33.3)	3 (75)
Nutrition^a^				
Plan required and not documented on discharge from ICU *n* (%)	24/53 (45.3)	52/132 (39.4)	76 (41.1)	8/14 (57)
Follow-up/outreach				
Seen by follow-up/outreach *n* (%)	53 (81.5)	154 (83.7)	207 (82.8)	15 (75)
Discharged *n* (%)	30 (56.6)	72 (46.8)	102 (49.3)	14 (93)
Day discharged med (IQR)	1 (1–2)	1 (1–2)	1 (1–2)	2 (1–2)
Not re-assessed *n* (%)	21 (70)	53 (73.6)	74 (72.5)	13 (93)

*EOLC* end-of-life care

^a^n = 185 requiring nutritional plan

^b^Number for whom this was clinically appropriate

## Discussion

Worldwide, post-ICU in-hospital mortality rates are high, ranging from 4 to 13% [1–3]. Most of these patients are discharged with curative intent [1]. The reasons why these patients die are poorly described. To the best of our knowledge, this is the first study using the SJR method to describe the patient population who die in hospital following ICU discharge.

Around 1/6th of post-ICU in-hospital deaths were discharged to the ward with an end-of-life care plan. In over a quarter of the remaining cases, death had some degree of avoidability. Death was probably avoidable in around 8% of discharges, when those discharged for end-of-life care were excluded (6.7% of all the post-ICU in-hospital deaths) rising to around 18% (or 14.7% of all post-ICU in-hospital deaths) using the more relaxed definition. In 2017–2018, the national case-mix programme reported 8272 deaths in hospital following discharge from adult general critical care units [4]. Our figures suggest 551 (95% CI 346–827) of these deaths were probably avoidable, rising to 1213 (95% CI 903–1578) cases using the more relaxed definition.

In total, 155/250 deaths were judged to be unavoidable despite the presence of problems in post-ICU ward care. Although not the focus of the study, this finding suggests there may be a problem with ICU triage. For some patients for whom survival was highly unlikely, an ICU admission may have prolonged suffering. Nearly half the (small number of) survivors studied were judged to have received poor care, suggesting substantial problems exist with providing good post-ICU care, regardless of the patient outcome.

Problems in care occurred disproportionately in the first 24 h following discharge from an ICU, suggesting focusing on improving safety in this period is important. Effective handover of care requirements between ICU and the ward requires identification of a clear plan for how these requirements will be met [24, 25]. The RCRR classifications of poor or very poor care occurred commonly in avoidable deaths, reflecting the complexity of care required. Importantly, all three organisations studied had average or above performance in the ICNARC Case Mix Programme during the period under study. Our findings therefore do not represent poor-performing institutions and so are likely to be generalisable.

### Comparison with previous work

Unlike previous work in entire hospital populations, we found no cases where death was definitely or strongly likely to have been avoidable. Conversely, 18% of deaths qualified for the more relaxed definition of avoidable, in comparison with 8.5% in general hospital populations [9]. Similarly, 26% of cases had some degree of avoidability, more than the 6.7% reported in general hospital populations [11]. Hogan et al. [9] classified 5.2% (95% CI 3.8–6.6) of deaths in the general hospital population they studied as avoidable, similar to Rogne et al. at 4.2% [11]. Although the numerical proportion in our post-ICU cohort was higher at 8% (95% CI 5.0–12.1), with a higher lower confidence limit, it remains possible that overall rates are similar.

It is possible the differences we found are explained by the population in our study. Inherent in having been to intensive care may be the understanding that there has been a risk of not surviving, making classifying subsequent death as entirely avoidable difficult. However, the complex care required by this post-ICU population may mean there are more aspects of care to be missed. We cannot exclude the possibility that the differences result from other differences between the studies.

Our study investigated the post-ICU care period. As problems in care may occur prior to ICU discharge, our findings may underestimate the overall in-hospital avoidability of death in this patient group. Further work is also required to investigate whether problems in care whilst in ICU contribute to adverse outcomes after ICU discharge.

Less than adequate care occurred rarely in patients where death was judged not avoidable, in common with previous work [9]. In contrast, less than adequate care occurred in over 70% of those with a problem contributing to mortality—double the rate seen in Hogan et al.’s previous study [9]. Whilst again this may suggest differences in the study rather than the population, it seems possible that our findings may reflect the difficulties presented to general wards in caring for the complex post-ICU population. In this cohort, non-survivors were considerably frailer at ICU admission than survivors (Table 1). Frail patients are known to be at higher risk of adverse events in hospital [26].

Out-of-hours discharge is highly associated with in-hospital post-ICU mortality and readmission [1, 2, 19]. In part this has been suggested to result from a higher proportion of patients with an end-of-life care plan in place being discharged at night [1]. However, we found discharge out-of-hours to be very common in patients who were not discharged for end-of-life care.

In this study, physical dependence at ICU discharge was high. Our findings are in line with a previous small study of patients who had spent 48 h or more on an ICU [27]. Perhaps because of the severity of dependency, delivering daily rehabilitation for this cohort occurred rarely on the ward, despite being essential to maximise physical recovery from critical illness [28, 29].

Both ongoing nutritional support needs and new episodes of sepsis were common post-ICU, with problems with ongoing management frequently identified. Previous studies have also demonstrated poor delivery of nutrition in post-ICU patients [30, 31] which has been linked to poor physical rehabilitation in the medium and long term [32, 33]. Sepsis is known to impair nutritional status and therefore may impact ongoing rehabilitation [34, 35]. In addition, new onset AF, also identified in this study as poorly managed, is a common complication of critical illness [36] where onset is known to be associated with sepsis and may be triggered by systemic inflammation [37].

Previous studies have suggested follow-up visits from specialist ICU outreach nurses may improve post-ICU survival and reduce adverse events [38–40]. However, in this study patients were frequently discharged from this service in the first 1–2 days following ICU discharge. There was no difference in the proportion of patients experiencing early discharge between possibly avoidable and unavoidable deaths, suggesting future opportunities to focus this service on those patients who would benefit the most from such visits.

Patients frequently had more than one problem in care [for example, over half the patients studied had severe mobilisation difficulties and over half developed sepsis, demonstrating that problems frequently overlapped (Table 5)]. Future interventions will need to address multiple needs to impact outcomes for these vulnerable patients.

### Strengths and limitations

Our study has several strengths. Data collection was undertaken by a small multi-disciplinary team. This provided a wider insight into care delivery than in other studies where reviewing teams consisted solely of medical staff. Reviewers also worked collaboratively, were able to communicate freely and discuss uncertainties in cases. We piloted our data collection forms and defined each of the variables collected in standard operating procedures. As a result, in undiscussed cases, our inter-rater reliability was at least as good as previous studies [9, 41, 42]. Our multi-centre design, with population descriptors mainly comparable to national data, suggests our findings should be generalisable, at least within the UK. Additionally, undertaking the same process on a small number of survivors helps place our findings in context.

However, several weaknesses should be acknowledged. RCRR relies on documentation from a variety of sources, which risks missing information due to omissions or inconsistencies in documentation. During the review process this was acknowledged. Care was taken to ensure all potential sources of documentation were carefully reviewed to assess actions in care delivery. In addition, 52/352 cases were excluded due to unavailable or incomplete records. This was likely inevitable due to the reliance on paper documents but may have introduced a degree of selection bias. It is also possible that documents in the included cases may have been missing but not detected where they did not form a clear part of the chronological record. As with all retrospective case record reviews, a problem of hindsight bias must be acknowledged [21, 43]. Knowledge of outcome severity has been shown to affect assessment of the quality of (anaesthetic) care [43]. However, it is not realistic to blind the reviewer from the (likely) outcome without removing key information for the analysis, and this has not been attempted in major studies in the field [9, 11].

Our sample size was larger than in similar studies of specific patient cohorts [44–46] but smaller than in previous work focused on general hospital populations, where a smaller proportion of deaths were anticipated to be modifiable [9, 11]. As a result, our estimates of the rates of avoidable deaths have relatively wide confidence intervals. However, we chose instead to record greater detail on specific pre-identified care issues to inform both clinicians and future work on how such deaths could be prevented. Our sample of 20 hospital survivors is small, as this was not a key focus of the overall REFLECT study. Importantly, it shows that problems in care are common post-ICU, regardless of outcome, but further work is needed to allow comparisons with other groups to be made.

Specific pre-identified care issues were chosen following literature review and findings from a previous audit. There may have been other problematic aspects of care, for which we did not collect quantitative data. We focused  our investigation on patients who did not survive, so cannot determine whether similar problems in care occur in those who survive to hospital discharge. As part of the overall REFLECT project, we will undertake in-depth analysis of the care received in those deaths judged to be avoidable in comparison with an equal number of patients who survived to address this issue.

## Conclusions

There is significant avoidability associated with death on the ward following ICU discharge. This avoidability occurs in an elderly frail population with complex needs that current strategies struggle to meet. Our work highlights opportunities to address common problems in care delivery which could improve both patient outcome and quality of care. Problems in care occurred disproportionately in the first 24 h following discharge from an ICU, suggesting interventions to improve safety should concentrate on this period. Recognition and management of sepsis, mobilisation and provision of nutrition were frequently sub-optimal and could be improved. Targeted CCOT input may assist in delivering these improvements but would require regular ward review beyond the first discharge day.

## Supplementary Information

**Additional file 1:** Additional data.

## Data Availability

The datasets used during the current study are available from the corresponding author on reasonable request.

## Abbreviations

AF: Atrial fibrillation
CCOT: Critical care outreach team
CFS: Clinical Frailty Score
EOLC: End-of-life care
ICNARC: Intensive Care National Audit and Research Centre
ICU: Intensive care unit
RCRR: Retrospective case record review
SJR: Structured Judgement Review

## References

[1] Santamaria JD, Duke GJ, Pilcher DV, Cooper J, Moran J, Bellomo R (2015). The timing of discharge from ICU and subsequent mortality: a prospective multi-center study. Am J Respir Crit Care Med.

[2] Goldfrad C, Rowan K (2000). Consequences of discharges from intensive care at night. Lancet.

[3] Araújo I, Gonçalves-Pereira J, Teixeira S, Nazareth R, Silvestre J, Mendes V, Tapadinhas C, Póvoa P (2012). Assessment of risk factors for in-hospital mortality after intensive care unit discharge. Biomarkers.

[4] ICNARC. Key statistics from the Case Mix Programme— adult, general critical care units Critical care units in the Case Mix Programme. 2018. https://www.icnarc.org/Our-Audit/Audits/Cmp/Reports/Summary-Statistics. Accessed 31 Jan 2020.

[5] Campbell A, Cook J, Adey G, Cuthbertson B (2008). Predicting death and readmission after intensive care discharge. Br J Anaesth.

[6] Elliott M, Worrall-Carter L, Page K (2013). Factors contributing to adverse events after ICU discharge: a survey of liaison nurses. Aust Crit Care.

[7] Denehy L, Skinner EH, Edbrooke L, Haines K, Warrillow S, Hawthorne G, Gough K, Vander Hoorn S, Morris M, Berney S (2013). Exercise rehabilitation for patients with critical illness: a randomized controlled trial with 12 months of follow-up. Crit Care.

[8] Walsh TS, Salisbury LG, Merriweather JL, Boyd J, Griffith D, Huby G, Kean S, Mackenzie S, Krishan A, Lewis S, Murray G, Forbes J, Smith J, Rattray J, Hull A, Ramsey P (2015). Increased hospital-based physical rehabilitation and information provision after intensive care unit discharge: the RECOVER randomized clinical trial. JAMA Intern Med.

[9] Hogan H, Healey F, Neale G, Thomson R, Vincent C, Black N (2012). Preventable deaths due to problems in care in English acute hospitals: a retrospective case record review study. BMJ Qual Saf.

[10] Hutchinson A, Coster JE, Cooper KL, Pearson M, McIntosh A, Bath PA (2013). A structured judgement method to enhance mortality case note review: development and evaluation. BMJ Qual Saf.

[11] Rogne T, Nordseth T, Marhaug G, Berg EM, Tromsdal A, Saether O, Gisvold S, Hatlen P, Hogan H, Solligard E (2019). Rate of avoidable deaths in a Norwegian hospital trust as judged by retrospective chart review. BMJ Qual Saf.

[12] Royal College of Physicians (2017). Using the structured judgement review methods: data collection form.

[13] Intensive Care Society. Guidelines for the Provision of Intensive Care Services (GPICS). 2019. https://www.ficm.ac.uk/standards-research-revalidation/guidelines-provision-intensive-care-services-v2. Accessed 31 Jan 2020.

[14] Vollam S, Gustafson O, Hinton L, Morgan L, Pattison N, Thomas H, Young JD, Watkinson P (2019). Protocol for a mixed-methods exploratory investigation of care following intensive care discharge: the REFLECT study. BMJ Open.

[15] Von Elm E, Altman DG, Egger M, Pocok SJ, Gotzsche PC, Vandenbroucke JP (2007). Strengthening the Reporting of Observational Studies in Epidemiology (STROBE) statement: guidelines for reporting observational studies. BMJ.

[16] Rockwood K, Song X, MacKnight C, Bergman H, Hogan DB, McDowell I, Mitnitski A (2005). A global clinical measure of fitness and frailty in elderly people. Can Med Assoc J.

[17] Knaus WA, Draper EA, Wagner DP, Zimmerman JE (1985). APACHE II: a severity of disease classification system. Crit Care Med.

[18] Royal College of Physicians (2016). Using the structured judgement review method: a clinical governance guide to mortality case record reviews.

[19] Vollam S, Dutton S, Lamb S, Petrinic T, Young JD, Watkinson P (2018). Out-of-hours discharge from intensive care, in-hospital mortality and intensive care readmission rates: a systematic review and meta-analysis. Intensive Care Med.

[20] Elliott M, Worrall-Carter L, Page KR, Vincent S (2012). Factors associated with in-hospital mortality following ICU discharge: a comprehensive review. Br J Intensive Care.

[21] Hutchinson A (2017). Using the structured judgement review method A guide for reviewers.

[22] R Development Core Team. 3.0.1. R: a language and environment for statistical computing; 2013.

[23] Daniels R, Nutbeam T, McNamara G, Galvin C (2011). The sepsis six and the severe sepsis resuscitation bundle: a prospective observational cohort study. Emerg Med J.

[24] Stelfox HT, Leigh JP, Dodek PM, Turgeon AF, Forster AJ, Lamontagne F, Fowler RA, Soo A, Bagshaw SM (2017). A multi-center prospective cohort study of patient transfers from the intensive care unit to the hospital ward. Intensive Care Med.

[25] Enger R, Andershed B (2018). Nurses’ experience of the transfer of ICU patients to general wards: a great responsibility and a huge challenge. J Clin Nurs.

[26] Bagshaw SM, Stelfox HT, McDermid RC, Rolfson DB, Tsuyuki RT, Baig N, Artiuch B, Ibrahim Q, Stollery DE, Rokosh E, Majumdar SR (2014). Association between frailty and short- and long-term outcomes among critically ill patients: a multicentre prospective cohort study. CMAJ.

[27] van der Schaaf M, Dettling DS, Beelen A, Lucus C, Dongelmans DA, Nollet F (2008). Poor functional status immediately after discharge from an intensive care unit. Disabil Rehabil.

[28] NICE. Rehabilitation after critical illness in adults: Quality standard 158. National London, UK: Institute for Health and Care Excellence; 2017.

[29] ICS (2019). Guidelines for the provision of intensive care services.

[30] Ridley EJ, Parke RL, Davies AR, Bailey M, Hodgson C, Deane AM, McGuiness S, Cooper DJ (2019). What happens to nutrition intake in the post-intensive care unit hospitalization period? An observational cohort study in critically ill adults. J Parenter Enter Nutr.

[31] Merriweather J, Smith P, Walsh T (2014). Nutritional rehabilitation after ICU—does it happen: a qualitative interview and observational study. J Clin Nurs.

[32] Dénes Z (2004). The influence of severe malnutrition on rehabilitation in patients with severe head injury. Disabil Rehabil.

[33] Wei X, Day AG, Ouellette-Kuntz H, Heyland DK (2015). The association between nutritional adequacy and long-term outcomes in critically ill patients requiring prolonged mechanical ventilation: a multicenter cohort study. Crit Care Med.

[34] Wischmeyer PE (2018). Nutrition therapy in sepsis. Crit Care Clin.

[35] McClave SA, Taylor BE, Martindale RG, Warren MM, Johnson DR, Braunschweig C, McCarthy MS, Davanos E, Rice TW, Cresci GA, Gervasio JM, Snacks GS, Roberts PR, Compher C, and the Society of Critical Care Medicine and the American Society for Parenteral and Enteral Nutrition (2016). Guidelines for the provision and assessment of nutrition support therapy in the adult critically ill patient. JPEN J Parenter Enter Nutr.

[36] Bosch NA, Cimini J, Walkey AJ (2018). Atrial fibrillation in the ICU. Chest.

[37] Meierhenrich R, Steinhilber E, Eggermann C, Weiss M, Voglic S, Bogelein D, Gauss A, Georieff M, Stahl W (2010). Incidence and prognostic impact of new-onset atrial fibrillation in patients with septic shock: a prospective observational study. Crit Care.

[38] Priestley G, Watson W, Rashidian A, Mozley C, Russell D, Wilson J, Cope J, Hart D, Kay D, Cowley K, Pateraki J (2004). Introducing critical care outreach: a ward-randomised trial of phased introduction in a general hospital. Intensive Care Med.

[39] Endacott R, Chaboyer W, Edington J, Thalib L (2010). Impact of an ICU Liaison Nurse Service on major adverse events in patients recently discharged from ICU. Resuscitation.

[40] Endacott R, Eliott S, Chaboyer W (2009). An integrative review and meta-synthesis of the scope and impact of intensive care liaison and outreach services. J Clin Nurs.

[41] Sorinola OO, Weerasinghe C, Brown R (2012). Preventable hospital mortality: learning from retrospective case record review. JRSM Short Rep.

[42] Sari ABA, Sheldon TA, Cracknell A (2007). Extent, nature and consequences of adverse events: results of a retrospective casenote review in a large NHS hospital. Qual Saf Health Care.

[43] Caplan RA, Posner KL, Cheney FW (1991). Effect of outcome on physician judgements of appropriateness of care. JAMA.

[44] Verlaat CW, van der Starre C, Hazelzet JA, Tibboel D, van der Hoeven J, Lemson J, Zegers M (2018). The occurrence of adverse events in low-risk non-survivors in pediatric intensive care patients: an exploratory study. Eur J Pediatr.

[45] Proulx N, Fréchette D, Toye B, Chan J, Kravcik S (2005). Delays in the administration of antibiotics are associated with mortality from adult acute bacterial meningitis. QJM.

[46] Siriwardena A, Akanuwe J, Crum A, Coster J, Jacques R, Turner J (2018). 20 Preventable mortality in patients at low risk of death requiring prehospital ambulance care: retrospective case record review study. BMJ Open.

