# Supplementary material for: Problems in care and avoidability of death after discharge from intensive care: a multi-centre retrospective case record review study
Source: Crit Care. 2021 Jan 6;25:10. doi: 10.1186/s13054-020-03420-5 (PMC7789328; doi:10.1186/s13054-020-03420-5)

# Additional Data File 1

### Table 1. Site characteristics

| Site | Hospital bed numbers | Number of ICU beds | Outreach/follow-up service |
| --- | --- | --- | --- |
| A | 1049 | 22 | ICU follow-up service 8am-6pm:  All patients with > 4 day ICU stay visited by follow-up nurses daily until deemed well enough to be discharged from the service. |
| B | 650 | 14 | ICU outreach service 24/7 and critical care rehabilitation service:  All patients with > 4 day ICU stay are visited by outreach nurses daily until deemed well enough to be discharged from the service. Additional weekly visit from the Rehabilitation after Critical Illness team. |
| C | 538 | 6 | ICU outreach service 24/7:  All patients > 48 hours ICU stay visited by outreach nurses daily until deemed well enough to be discharged from the service. |

### Table 2. Definitions, sources and rules for demographic variables

| Variable | Source | Interpretation rules |
| --- | --- | --- |
| Age at ICU discharge* † | Recorded on ICU discharge documentation or calculated from date of birth | n/a |
| Sex* † | Record in medical record | n/a |
| APACHE II* † | Electronic database | n/a |
| Admission diagnosis* † | ICU admission form | Surgical – required surgery prior/during ICU admission  Medical – no surgery required  Trauma – admitted with trauma-related problem |
| Type of admission* † | Medical notes | n/a |
| Clinical Frailty Score* † | Interpreted from multiple sources in medical notes, such as admission clerking, ICU nurse assessment, physio assessment. | Selection of score best fitting information recorded. Score selection verified from at least two sources of information. |
| Length of ICU/hospital stay* †  Days to death | Recorded in medical record (electronic or paper) | n/a |
| Palliative discharge | Medical notes  ICU discharge documentation | Clear plan for palliative care (palliative care team not always involved). |

*Data for survivors extracted from local site ICNARC databases

† Data for national cohort of non-palliative discharges extracted from central ICNARC database

### Table 3. Definitions, sources and rules for variables in pre-identified care issues

| Variable | Source | Interpretation rules |
| --- | --- | --- |
| Time of discharge from ICU | ICU documentation  Ward nursing documentation  Documentation of first observations on ward | 1^st^: ward nurse stated time of admission to ward  2^nd^: Time stated on ICU discharge summary  3^rd^: First observation set on ward  4^th^: Interpretation of category of time of day from documentation, e.g. morning, afternoon, early evening, night. |
| Mobility at ICU discharge | ICU physiotherapy documentation  ICU discharge documentation | If score not recorded by physiotherapist, interpretation from ICU documentation. |
| Mobilised to chair | Physiotherapy and nursing documentation | Yes = mobilised to chair on every day that patient condition allowed: as per physiotherapy and nursing documentation where present, or interpretation of patient condition using clinical judgement. |
| Mobilised away from bedspace | Physiotherapy and nursing documentation | Yes = mobilised away from bedspace on every day that patient condition allowed: as per physiotherapy and nursing documentation where present, or interpretation of patient condition using clinical judgement. |
| New diagnosis of AF | Medical notes | AF documented on ward and not documented in PMH or ICU discharge summary. |
| AF appropriate initial management | Medical notes, nursing notes, drug chart | Administration of any rate-controlling medication or consultation with cardiologist. |
| AF investigation of underlying cause | Medical notes | Documented investigation of any potential cause following onset of AF (not necessarily related to AF). For example, septic screen, CT scan to investigate potential septic sources, review and correction of electrolyte imbalance. |
| Sepsis: diagnosis/suspicion | Medical notes | Evidence of infection and documentation of sepsis diagnosis or suspicion  Documentation must include word ‘sepsis’. |
| Sepsis 6 completed  All aspects of sepsis 6 completed or considered:  a) Administer oxygen (or SpO_2_ above 94%)  b) Take blood cultures  c) Give IV antibiotics  d) Give IV fluids (if hypotensive or plasma lactate concentration >2mmol/l)  e) Check (serial) lactate concentration.  f) Measure urine output | Medical and nursing documentation, laboratory data, drug chart, fluid balance chart | a) Oxygen saturations of >94% on vital signs chart; oxygen administered if saturations below 94% in nursing notes  b) Documentation in medical notes of cultures taken; cultures documented in laboratory tests  c) Documentation in medical notes of antibiotic prescription; antibiotic prescription on drug chart  d) Normotension on vital signs chart; documentation of IV fluids given in nursing notes; documentation on fluid balance chart of IV fluid bolus  e) Lactate measurement documented in medical notes; arterial or venous blood gas result documented  f) Urine output documented on fluid balance chart |
| Nutrition plan required and completed | ICU discharge summary  Nutrition team documentation  Nursing documentation | Patient receiving enteral or parental feeding and/or  Requiring oral nutritional supplements and/or  Documented poor oral intake/need help to eat  AND  Documented plan from nutrition team |
| Seen by outreach/follow up | Medical notes | Documentation of outreach/follow-up visit on ward |
| Discharged from outreach/follow-up | Medical notes | Clear documentation of discharge (if not seen but no documentation of discharge = no) |
| Day discharged | Medical notes | n/a |
| Not reassessed | Medical notes  Nursing notes | Documentation of review following clinical deterioration after discharge from service |

###

### Table 4. RCRR agreement scores

|  | **First 24 hours** | **Ongoing management** | **Care during a procedure** | **End-of-life care** | **Overall Assessment** | **Avoidability of death** | **Overall** |
| --- | --- | --- | --- | --- | --- | --- | --- |
| Kappa (95% CI) | 0.70 (0.35-1.00) | 0.87 (0.53-1.00) | 1 (n/a-n/a)* | 0.42 (0.05-0.74) | 0.78 (0.44-1.00) | 1 (n/a-n/a)* | 0.77 (0.64-0.88) |

*CI n/a as all scores agreed

### Table 5. Reasons deaths judged to be unavoidable

| Reason death deemed unavoidable | Proportion of deaths with no avoidability  *n* = 185 |
| --- | --- |
| Progression of a known chronic disease | 51 |
| Made palliative within 24 hours of ICU discharge | 14 |
| Died suddenly within 48 hours of ICU discharge with no problems in care delivery | 5 |
| Had problems in care but deaths were deemed unavoidable | 64 |
| Had no problems in care delivery | 51 |

###

### Figure 1. Patient flow diagram

ICU Admissions

January 2015 – March 2018

N = 8,731

Site A = 4,798

Site B = 2,711

Site C = 1,222

Palliative discharge from ICU

N = 50

Site A = 60

Site B = 100

Site C = 140

Incomplete record = 16

Notes unavailable = 36

Died on ICU = 1,160

Discharged to other institution = 137

Identified deaths

N = 352

RCRR completed

N = 300

Non-palliative

N = 250

Discharged from ICU

N = 7,434

Survived to hospital discharge = 7082

### Figure 2. Time to death curve


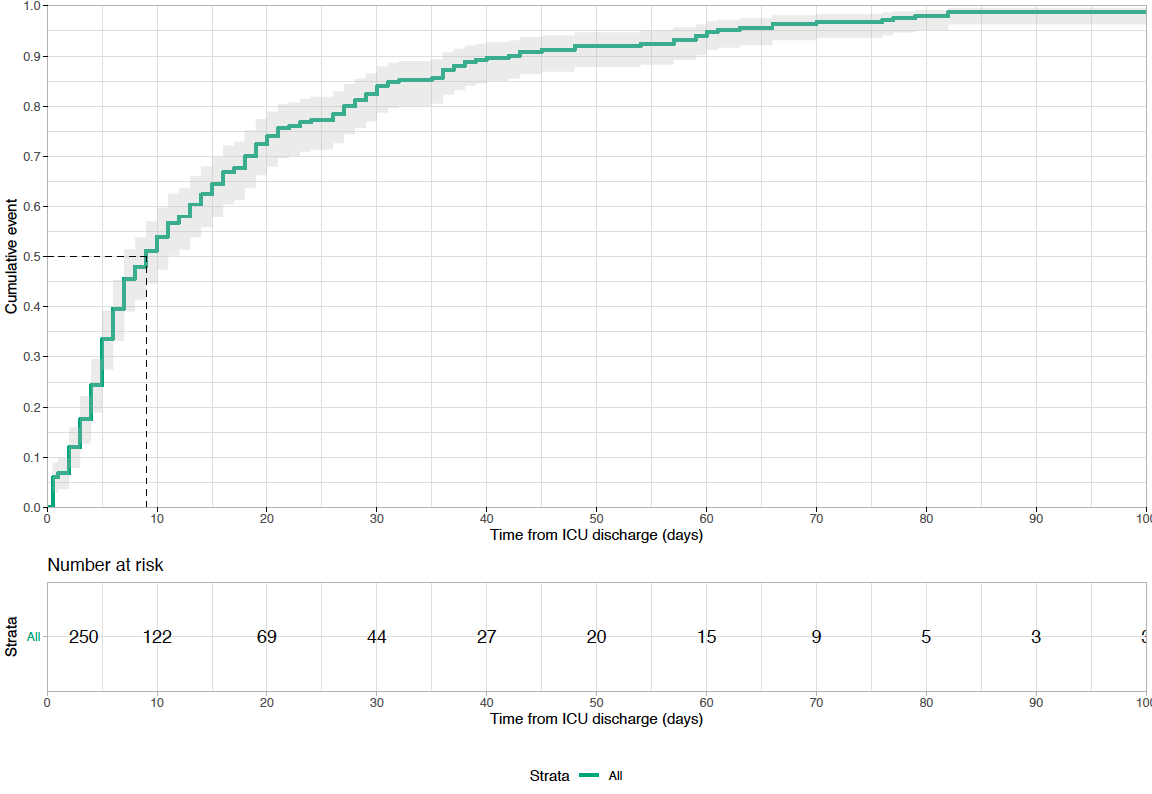

Supplement: Supplementary file 1 — Additional file 1: Additional data. [file 13054_2020_3420_MOESM1_ESM.docx]
